# Individualised Transcranial Magnetic Stimulation Targeting of the Left Dorsolateral Prefrontal Cortex for Enhancing Cognition: A Randomised Controlled Trial

**DOI:** 10.3390/brainsci14040299

**Published:** 2024-03-22

**Authors:** Donel M. Martin, Yon Su, Ho Fung Chan, Victoria Dielenberg, Esther Chow, Mei Xu, Ashley Wang, Stevan Nikolin, Adriano H. Moffa, Colleen K. Loo

**Affiliations:** 1 Discipline of Psychiatry and Mental Health, School of Clinical Medicine, Faculty of Medicine and Health, University of New South Wales, Sydney, NSW 2052, Australia; 2 Black Dog Institute, Sydney, NSW 2031, Australia

**Keywords:** transcranial magnetic stimulation, targeting, dorsolateral prefrontal cortex, cognition, healthy, cognitive flexibility

## Abstract

Repetitive transcranial magnetic stimulation (rTMS) has been demonstrated to produce cognitive enhancing effects across different neuropsychiatric disorders; however, so far, these effects have been limited. This trial investigated the efficacy of using a novel individualised approach to target the left dorsolateral prefrontal cortex (L-DLPFC) for enhancing cognitive flexibility based on performance on a cognitive task. First, forty healthy participants had their single target site at the L-DLPFC determined based on each individual’s performance on a random letter generation task. Participants then received, in a cross-over single-blinded experimental design, a single session of intermittent theta burst stimulation (iTBS) to their individualised DLPFC target site, an active control site and sham iTBS. Following each treatment condition, participants completed the Task Switching task and Colour–Word Stroop test. There was no significant main effect of treatment condition on the primary outcome measure of switch reaction times from the Task Switching task [F = 1.16 (2, 21.6), *p* = 0.33] or for any of the secondary cognitive outcome measures. The current results do not support the use of our novel individualised targeting methodology for enhancing cognitive flexibility in healthy participants. Research into alternative methodological targeting approaches is required to further improve rTMS’s cognitive enhancing effects.

## 1. Introduction

Cognitive flexibility is a higher-level ‘executive’ cognitive function and refers to a person’s ability to appropriately adjust their behaviour within a changing environment [1]. This ability plays an important role for academic achievement [2], social cognition [3], and resilience [4]. It is also critical for effective emotion regulation [5], which is dysfunctional in multiple mental health conditions such as Major Depressive Disorder (MDD [6]) and Borderline Personality Disorder [7]. Impaired cognitive flexibility is a defining characteristic of several neuropsychiatric conditions (e.g., autism spectrum disorder, obsessive-compulsive disorder: OCD) [8] and a common consequence of acquired brain injury (i.e., stroke, traumatic brain injury) [9]. In people with neuropsychiatric disorders, poor cognitive flexibility has been associated with reduced day-to-day functioning, including for work, relationships, and community interaction [10,11]. Currently, there is no effective treatment for people with impaired cognitive flexibility.

Transcranial magnetic stimulation (TMS) is a non-invasive form of brain stimulation that uses magnetic pulses to modulate neural activity and cognitive functions [12,13]. In a meta-analysis of randomised sham-controlled trials conducted in people with MDD, we observed that a therapeutic course of repetitive TMS (rTMS) administered to the dorsolateral prefrontal cortex (DLPFC) significantly improved performance on measures of cognitive flexibility (Hedge’s g = 0.26) and attentional processing speed (Hedge’s g = 0.28), although not for other individual cognitive measures [14]). This finding was consistent with recent meta-analyses conducted in other clinical populations indicating cognitive enhancing effects of rTMS (e.g., Alzheimer’s disease and mild cognitive impairment [15]; schizophrenia [16]; neuropsychiatric disorders [17]).

When high-frequency rTMS (HF-rTMS) is administered ‘online’, i.e., concurrently during the performance of a cognitive task, cognitive performance has been found to be decreased [12]. These impairing effects of ‘online’ HF-rTMS may be due to the introduction of random neuronal firing during ongoing cognitive processing [12]. Alternatively, when HF-rTMS is administered before the performance of a cognitive task (i.e., ‘offline’), performance has been shown to be improved [13,18]. A recent meta-analysis in healthy participants found that for the executive function domain and for all cognitive domains collapsed, ‘offline’ active HF-rTMS was associated with significantly better accuracy and reaction time performance compared to sham HF-rTMS [13]. However, the magnitude of the effect sizes for these cognitive enhancing effects of rTMS was small, and the between-study heterogeneity was high, indicating that enhanced ‘offline’ HF-rTMS protocols are required to further develop rTMS as a novel intervention for cognitive impairment.

The effectiveness of rTMS for modulating brain functioning and producing therapeutic and cognitive effects is limited by the accuracy of coil positioning upon the scalp. For other applications of rTMS, individualising the site of stimulation has been associated with enhanced therapeutic effects, for example, in treating MDD [19,20]. This is because rTMS’s neuromodulatory effects are typically highly focal (i.e., approximately 1.5 cm diameter, 2 cm depth). For enhancing cognition, the left DLPFC (L-DLPFC) has been the target site of most research interest [13,15,18,21]. The L-DLPFC is a highly linked node in the central executive network that is associated with mood regulation and higher-level cognitive processes including working memory [22] and cognitive flexibility [23]. However, despite the widespread use of the L-DLPFC as a target for rTMS for cognition, the optimal methodology for targeting this region for cognitive enhancement remains unclear.

When administering rTMS, the treatment site on the head is typically determined using one of several different standardised methods. These include using a standard position based on the International 10–20 EEG system or standardised placements based on group-MRI structural [24] or resting-state functional scans [25]. Interestingly, the use of these different standardised methods usually leads to variability in the target region and may produce placements on the same individual’s head that can vary by several centimetres [26]. Importantly, when applied to targeting rTMS to the DLPFC to enhance cognition, none of these standardised methods are based on an individual’s brain functioning during a cognitive task. Recent research has investigated the use of functional neuroimaging (fMRI) during the performance of a cognitive task to individualise the site of ‘online’ rTMS (e.g., [27]), though results remain inconclusive.

In a recent novel proof-of-concept study in healthy participants, we investigated whether a behavioural measure (i.e., a cognitive task) alone could be used to assist with individualising the site of stimulation for rTMS over the L-DLPFC [28]. The advantage of using performance outcomes alone meant that neuroimaging was not required, unlike in other individualised structural- and functional-based targeting methods [29]. The methodology involved administering ‘online’ rTMS to different sites at the L-DLPFC during the performance of a random letter generation task, which assessed cognitive flexibility. Neuroimaging studies have identified the L-DLPFC as an important brain region for subserving cognitive flexibility [30,31]. An inhibitory ‘online’ rTMS protocol was utilised during task performance, as this has previously been demonstrated to reduce cognitive flexibility function [23,31]. Our results confirmed that active ‘online’ rTMS administered over the L-DLPFC could modify (i.e., reduce) performance on the task compared with sham rTMS. Further, a single individualised site of stimulation based on performance outcomes was identified in the majority of participants [28]. These results provided a proof of concept for a method to individualise the site of stimulation for rTMS at the L-DLPFC for modulating and improving cognitive flexibility.

In this randomised sham-controlled trial, we first applied this novel targeting method (i.e., ‘online’ rTMS during cognitive task performance) to identify each individual’s optimal stimulation site at the L-DLPFC and then prospectively tested the efficacy of targeting this identified site for improving cognitive flexibility performance following a single session of ‘offline’ active or sham HF-rTMS. To the best of our knowledge, this was the first randomised controlled trial to investigate the efficacy of a single session of ‘offline’ HF-rTMS administered to an individualised target site and an active control site at the L-DLPFC for enhancing cognitive flexibility. Outcomes from this study are expected to assist with informing the efficacy of individualised targeting of the L-DLPFC with HF-rTMS for enhancing cognitive functioning.

## 2. Methods

### 2.1. Participants

Inclusion was restricted to healthy subjects who were right handed as assessed by the Edinburgh Handedness Inventory [32] and aged between 18 and 40 years. Exclusion criteria were as follows: currently taking concurrent TMS-effecting medications (e.g., benzodiazepines), recent head injury, current pregnancy, history of seizure or stroke, any psychiatric or neurological illness, any serious medical conditions, or current history of drug or alcohol abuse or dependence. The study was powered to detect at least a moderate difference of d = 0.5 between the individualised active target site and sham rTMS stimulation with a total sample size of N = 34 (alpha = 5%, power = 80%). The study aimed to recruit 40 participants to account for potential dropouts. The study was approved by the Human Research Ethics Committee of the University of New South Wales, and all participants provided written informed consent. The clinical trial was registered with the Australian New Zealand Clinical Trials Registry: ACTRN12620000162910p.

### 2.2. Study Design

The study utilised a within-subject, randomised, sham-controlled, single-blinded experimental design. Prior to the commencement of recruitment, a randomisation list was computer-generated by a study investigator (V.D.), and this counterbalanced the order of sites for the administration of the active and sham TMS conditions (e.g., central, lateral, sham, anterior, posterior, medial) for Session 1 and the order of the different stimulation conditions for the experimental conditions for Sessions 2 to 4. Recruited participants were sequentially allocated by a study investigator (V.D. or H.F.C) to a particular randomisation sequence for both Session 1 and Sessions 2 to 4. Only the study investigators who transcribed, scored, and analysed the performance outcomes from Session 1 and the participants were blinded.

**Figure 1:**
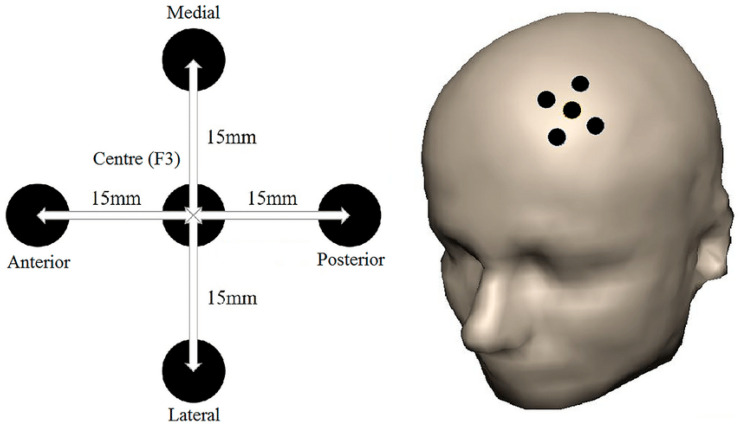
Five sites over the left dorsolateral prefrontal cortex (L-DLPFC), which were targeted by our novel methodology to determine the individualised target site in Session 1. Notes: F3 is located using a standard International 10–20 electroencephalography (EEG) cap and marked. A common template was applied to mark sites 1.5 cm anterior, posterior, medial, and lateral to F3. Figure has been taken from “A novel approach for targeting the left dorsolateral prefrontal cortex for transcranial magnetic stimulation using a cognitive task.” by Wang et al. [28] (https://doi.org/10.1007/s00221-021-06233-2 (accessed on 8 October 2021).

Participants first completed an initial experimental session (Session 1) where they received active or sham ‘online’ TMS administered to five different locations at the L-DLPFC (F3 according to the International 10/20 EEG system; see Figure 1) during performance of a random letter generation task over 8 trial blocks [28]. The five stimulation sites were first marked on the scalp using a custom-printed template with the anterior and posterior sites aligned parallel to the participant’s midsagittal plane. The five sites were positioned at F3 and 1.5 cm medially, anteriorly, laterally, and posteriorly around the F3 site, consistent with our prior study [28]. Active ‘online’ rTMS was then administered during performance of the random letter generation task to each of the 5 sites in addition to a sham rTMS block following the first two blocks that involved no stimulation [28]. The sequential order of sites for administration of active or sham rTMS was according to each participant’s randomised order. The duration of each task block was 2 min, with short breaks provided in between, during which the coil was repositioned. Our targeting methodology has been described in detail elsewhere [28]. The purpose of this first experimental session was to determine both an individualised active target site and an individualised active control site based on participants’ performance on the random letter generation task. Each participant’s individualised active site was defined as the single site that had the highest sum rank out of 10 (with a total rank-sum score of at least 8/10) of the difference between the total number of unique digram (i.e., unique combinations of letter pairs, e.g., AB) and trigram (i.e., unique combinations of letter triplets, e.g., ABC) scores compared to the sham condition based on performance on the random letter generation task. Each participant’s active control site was defined as the site with the lowest total rank-sum score. In the case of a tie in rank sums between two sites, the individualised target or control site was determined as the first of the two tied sites administered according to that participant’s randomised sequence order of rTMS over the 5 active rTMS sites during Session 1.

**Figure 2:**
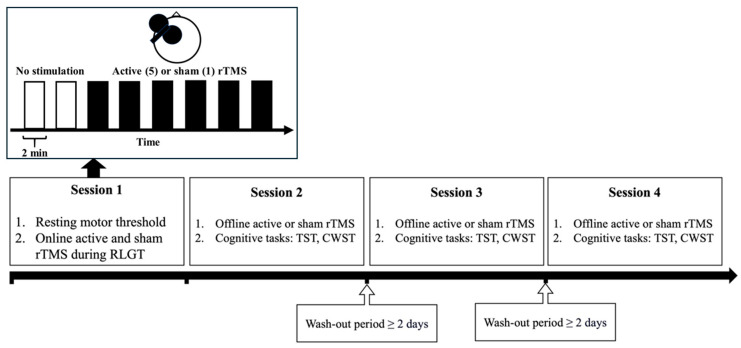
Study design. Notes: rTMS is repetitive transcranial magnetic stimulation, RLGT is the random letter generation task, TST is the Task Switching Task, and CWST is the Colour–Word Stroop Test. During Session 1, participants completed the RLGT during ‘online’ active and sham rTMS for each of the 5 targeted stimulation sites at and around F3 (International 10–20 EEG system). Following Session 1, participants completed Sessions 2–4 in a randomised counterbalanced order where they received ‘offline’ active rTMS administered to an individualised target site (N = 6 at a central (F3) site, N = 8 at a medial site, N = 9 at an anterior site, N = 6 at a lateral site, and N = 4 at a posterior site) and the active control site (N = 10 at a central site, N = 10 at a medial site, N = 3 at an anterior site, N = 4 at a lateral site, and N = 6 at a posterior site) and sham rTMS prior to performing both the TST and CWST.

Following this, participants completed three experimental sessions (Sessions 2–4) in a randomised, counterbalanced order, with each session conducted at least two days apart from the previous session (no maximum time between sessions). Prior to receiving ‘offline’ rTMS at each session, participants practiced both cognitive tasks. During each of Sessions 2 to 4, participants received either a single session of active rTMS, administered to the individualised target site or to their active control site, or sham rTMS. Following the administration of active or sham rTMS, participants completed two cognitive tasks (the Task Switching Task and then the Colour–Word Stroop Task), starting 10 min following the end of stimulation [33,34]. The trial design is shown in Figure 2 below.

### 2.3. Transcranial Magnetic Stimulation

TMS was administered using a Cool-B65 ‘figure-of-8’ coil and a MagPro X100 stimulator (MagVenture Company, Lucernemarken, Denmark). This coil has higher focality and electric field depth decay relative to other commercially available coils [35]. A study that investigated the focality of a figure-eight coil stimulation effects over the motor cortex indicated a functional spatial resolution of 5 mm [36]. Participants had their resting motor threshold (RMT) determined by titrating the intensity of the TMS stimulus to obtain the minimal stimulus intensity required to elicit 3 of 6 consecutive motor-evoked potentials with a peak-to-peak amplitude of at least 50 μV using electromyography (EMG). TMS was administered using a ‘figure-of-8’ coil to ensure the focality of stimulation. The coil was positioned with the handle facing posterolaterally at a 45° angle to the participant’s midline. During experimental Session 1, active ‘online’ rTMS involved delivering a train of 4 magnetic pulses at 20 Hz at 110% RMT stimulus intensity 300 ms before the onset of responses on alternate task trials (i.e., prior to 50/100 test trials) on the random letter generation task. For sham rTMS, the coil was similarly placed on the head, though no active stimulation was administered. For this condition, an identical active rTMS protocol was simultaneously triggered in a separate TMS coil located nearby to control for placebo and auditory effects. For this experimental session, the current direction of the coil was reversed using the Magventure stimulator to produce a biphasic stimulus, which resulted in a predominantly anterior-to-posterior induced current consistent with Jahanshahi and Dirnberger [23]. For experimental Sessions 2 to 4, ‘offline’ active intermittent theta-burst stimulation (iTBS) was administered for 190 s (600 pulses) at 110% RMT. For sham rTMS, the active coil was similarly disconnected and placed on the head over the L-DLPFC (F3), and a second connected coil was placed behind the head and activated to mimic the auditory ‘clicking’ of active iTBS. This sham condition was designed to control for the placebo effect of rTMS and auditory effects whilst also eliminating any potential neural effects from stimulation. For experimental Sessions 2–4, the current of the coil was changed to produce a predominantly posterior-to-anterior induced current using the biphasic stimulus, consistent with prior studies targeting this region for cognitive enhancement with active ‘offline’ iTBS (e.g., [37]).

### 2.4. Random Letter Generation Task

The task was based on that described by Jahanshahi and Dirnberger [23] and chosen as a measure of cognitive flexibility in the context of the strongly stereotyped order of the English alphabet. The task was administered through custom-made scripts using the Inquisit software package (Version 5, Millisecond Software LLC, Seattle, DC, USA). During Session 1, participants were required to say letters between A and I in a random sequence using the ‘hat’ analogy [23] while receiving ‘online’ active or sham rTMS. This ‘hat’ analogy is as follows: ‘As an example of the concept of randomness, suppose we had written the letters A through to I on separate pieces of paper and put them into a hat. You take out one piece of paper, call out the letter on it, e.g., “C”, and return it to the hat. Then you would reach for another piece of paper and do the same thing, e.g., “F”. The series of letters you would call out in that way would be random’. Participants had to synchronise their responses to a visual stimulus of a white circle presented against a black background. The stimulus was presented at a rate of once every 1.2 s over 100 trials for each of the 8 trial blocks. Participants’ verbal responses for each of the 8 blocks were recorded and then transcribed and scored by an investigator who was blinded to each of the stimulus conditions during Session 1 (E.C and H.F.C). The outcome measures were the numbers of unique digrams and trigrams achieved in each block of 100 letters. The maximum possible number of unique digrams was 81, and for unique trigrams, this number was 98.

### 2.5. Task Switching Task

Both cognitive tasks for experimental Sessions 2 to 4 were administered using E Prime 2.0 (Psychology Software Tools, Sharpsburg, PA, USA). The Task Switching Task was used to assess cognitive flexibility due to its sensitivity to rTMS administered to the L-DLPFC [38]. Participants were presented with a letter and a number (e.g., ‘G5’) inside a coloured square. They were instructed to name the letter if the square was blue and name the number if the square was yellow. The colour of the square switched every 2 stimuli. Response times for correct responses were measured using an SV-1 Voice Key (Cedrus Corporation, San Pedro, CA, USA). The primary outcome measure was reaction time for correct switch trials, with reaction time for non-switch trials as a secondary outcome. There were 63 switch and 64 non-switch trials. The total duration of the task was approximately 10 min.

### 2.6. Colour–Word Stroop Task

The Colour–Word Stroop task was administered to assess cognitive inhibition, a component of cognitive flexibility and key executive function [39]. Prior studies have demonstrated cognitive enhancement on the Stroop following stimulation of the L-DLPFC with HF-rTMS in healthy participants [40,41]. Participants were presented with the name of a colour displayed in a certain ink colour, which was either congruent or incongruent with the meaning of the word. Participants responded by indicating the colour of each word’s lettering regardless of its meaning. Response times for correct responses were measured using an SV-1 Voice Key (Cedrus Corporation, San Pedro, CA, USA). The outcome measures were reaction times to correctly identify ‘incongruent’ and ‘congruent’ ink colours. There were 96 congruent and 48 incongruent trials. The duration of the task was approximately 5 min.

### 2.7. Statistical Analysis

Statistical analyses were performed using the Statistical Package for the Social Sciences (SPSS) for Windows (Version 26.0, IBM Corp., Armonk, NY, USA). For the primary and secondary cognitive outcome measures, individual trials on each task were excluded for RTs < 200 ms. Primary and secondary outcomes were analysed for participants with cognitive data for experimental Sessions 2 to 4. Data were analysed using a mixed-effects repeated-measures model, with the within-subject factor being condition (two active rTMS conditions and sham rTMS) and subject as a random factor. Mixed-effects repeated-measures models have the advantages of being able to model individual subject effects and can handle missing data at specific time points. Secondary sensitivity analyses were conducted using the same statistical models, excluding participants with >20% of data missing for a test outcome measure due to ineligible responses/or equipment errors using the Voice Key data recorder. Statistical significance was set at *p* < 0.05.

## 3. Results

### 3.1. Participants

Forty participants were screened and randomised. Seven participants subsequently withdrew: four due to COVID-19-related pauses in the study, two participants because they could not tolerate rTMS in experimental Sessions 2 to 4, and one due to unknown reasons. Data from 33 participants were analysed for the per-protocol analysis. For the sensitivity analysis, data from nine sessions (N = 5 participants) for the Task Switching Task were excluded, and for the Colour–Word Stroop task, data from three sessions were excluded (N = 3). The mean age of participants was 23.1 (4.14) years, with 16 males/17 females and an average of 16.2 (1.75) total years of education. For the individualised target sites determined from Session 1 for the single active experimental session (i.e., one of Sessions 2 to 4), N = 6 had a central (F3) site, N = 8 had a medial site, N = 9 had an anterior site, N = 6 had a lateral site, and N = 4 had a posterior site. For the active control site, N = 10 had a central site, N = 10 had a medial site, N = 3 had an anterior site, N = 4 had a lateral site, and N = 6 had a posterior site.

### 3.2. Task Switching Task

**Figure 3:**
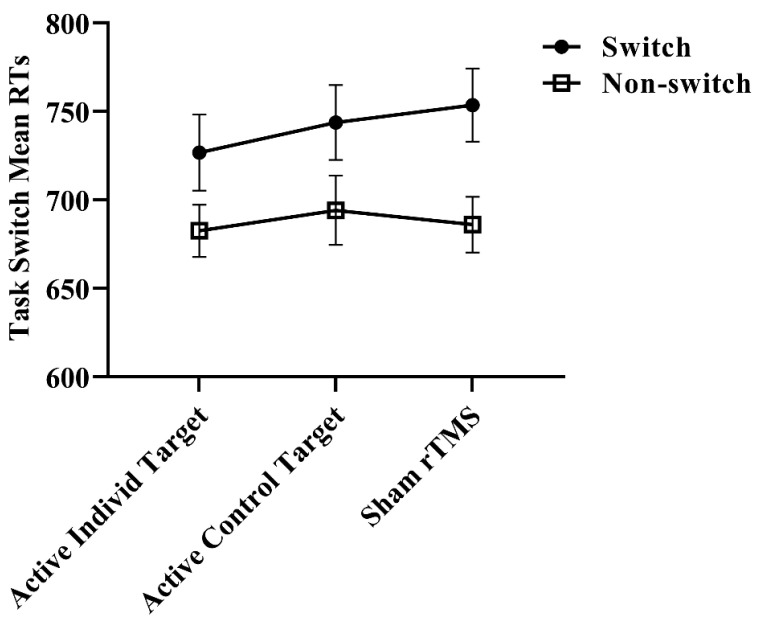
Performance outcomes on the Task Switching Task for the primary and secondary outcome measure following ‘offline’ iTBS. Error bars represent the standard errors of the mean (SEMs). RTs: reaction times; rTMS: repetitive transcranial magnetic current stimulation.

For the primary outcome measure (i.e., the switch condition), there was no significant main effect of condition [F = 1.16 (2, 21.6), *p* = 0.33; see Figure 3]. For the secondary outcome (i.e., the non-switch condition), there was similarly no significant main effect of condition [F = 0.23 (2, 38.7), *p* = 0.80; see Figure 3]. These results remained unchanged with the sensitivity analysis: switch [F = 0.40 (2, 25.8), *p* = 0.68] and non-switch [F = 0.22 (2, 26.4), *p* = 0.81].

### 3.3. Colour–Word Stroop Task

**Figure 4:**
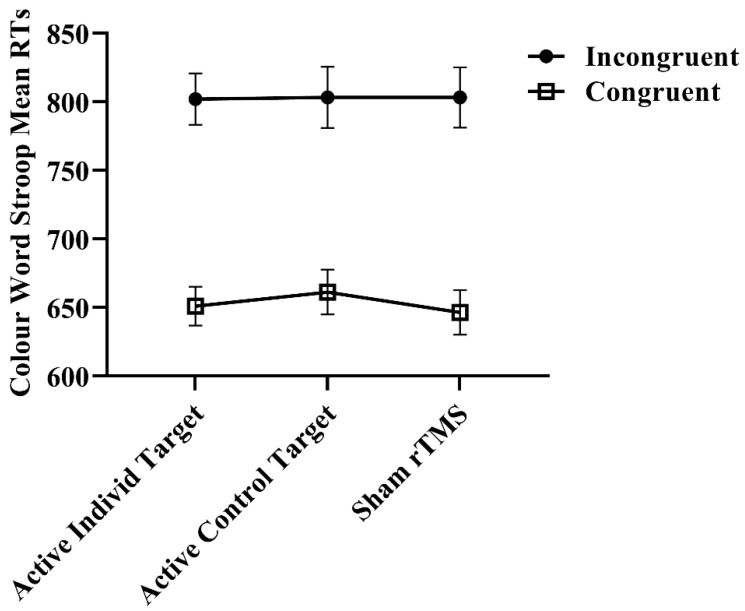
Secondary cognitive outcomes on the Colour–Word Stroop Task following ‘offline’ iTBS. Error bars represent SEMs. RTs: reaction times; rTMS: repetitive transcranial magnetic current stimulation.

For the secondary cognitive outcomes on the Colour–Word Stroop Task, there was no significant main effect of condition for the incongruent condition [F = 0.01 (2, 41.1), *p* = 1.0; see Figure 4] or for the congruent condition [F = 0.54 (2, 40.2), *p* = 0.59; see Figure 4]. These results remained unchanged with the sensitivity analysis: incongruent [F = 0.44 (2, 31.8), *p* = 0.65] and congruent [F = 0.44 (2, 34.6), *p* = 0.65].

### 3.4. Assessment of Safety

From the 99 experimental sessions involving active or sham iTBS, mild to moderate muscle twitching was reported in 15% of sessions, scalp discomfort in 11% of sessions, eye discomfort in 4% of sessions, jaw clenching and dizziness in 3% of sessions, and mild headache and sleepiness in 1% of sessions. The muscle twitching, scalp discomfort, eye discomfort and jaw clenching were all transient side effects that occurred either during or immediately after rTMS. All side effects were resolved within 24 h following the cessation of the experimental sessions.

## 4. Discussion

This study investigated whether the use of a novel individualised targeting methodology involving ‘online’ rTMS and a behavioural measure could improve cognitive flexibility performance following a single session of ‘offline’ iTBS administered to the L-DLPFC in healthy participants. Contrary to our hypothesis, no significant differences between stimulation conditions were found for the primary and secondary cognitive outcome measures.

While previous ‘online’ rTMS studies have demonstrated the role of the L-DLPFC in underlying cognitive flexibility [23], few studies have investigated the effects of ‘offline’ rTMS administered to this region for modulating cognitive flexibility performance. In people with major depression receiving twice-daily rTMS treatment administered to the L-DLPFC, Aoun and colleagues [38] administered the Task Switching Task at baseline and again after six, twelve, and thirty sessions, with response times recorded with a voice key, similarly to the current study. Results showed that remitters had significantly better cognitive flexibility at pre-treatment and at post-treatment, and significantly improved in the switch reaction time from baseline at the subsequent assessments while there was no significant change in performance for non-remitters. This suggested that patients with better cognitive flexibility at baseline may show greater clinical response to rTMS and potentially also greater improvement in cognitive flexibility with treatment.

In healthy participants, two prior sham-controlled studies have examined the acute cognitive enhancing effects of a single session of ‘offline’ HF-rTMS administered to the L-DLPFC using different cognitive flexibility tasks. Huang et al. [42] investigated the effects of a single session of offline 5 Hz rTMS (1600 pulses) on response inhibition using a Go/No Go task and found no effect on either accuracy or reaction time following stimulation. Pinto et al. [43] administered a single session of offline iTBS (600 pulses) over the L-DLPFC and similarly reported no significant performance effects on either the Trail Making Test or Colour–Word Stroop Test following stimulation. In those studies, the L-DLPFC site was identified by the site 5 cm anterior to the site of maximum activation of the right abductor polis brevis (APB) muscle. In the current study, we instead targeted an individualised site at or around F3 on the International 10–20 EEG system with a single session of active ‘offline’ iTBS. In addition, we investigated cognitive effects following a single session of active ‘offline’ iTBS administered over an active control site in this same region. Consistent with those prior findings, the current results showed no significant performance-enhancing effects following a single session of ‘offline’ iTBS administered to either an individualised site at the L-DLPFC or over an active control site also over this region. Taken together, these results suggest that a single session of ‘offline’ iTBS administered over the L-DLPFC has minimal or no cognitive enhancing effects for cognitive flexibility in healthy participants. This is consistent with recent reviews showing limited cognitive effects of rTMS administered to the L-DLPFC [18] and/or other brain regions in healthy participants [13].

Strengths of this study included the investigation of a novel individualised targeting approach to improve the cognitive efficacy of ‘offline’ iTBS, the inclusion of an active control site, and a randomised sham-controlled experimental design. Potential limitations included the use of healthy subjects, which may have limited the potential for observing cognitive enhancing effects due to intact cognitive functioning and produced ceiling effects with task performance. Future studies investigating novel methodological approaches for improving the cognitive efficacy of rTMS may benefit from using clinical samples with cognitive dysfunction or impairment. Due to the limited cognitive effects of a single session of ‘offline’ rTMS [13], future studies may additionally consider investigating the effects of repeated treatments.

While research in both healthy and clinical samples currently indicates that rTMS has cognitive enhancing effects, additional research is required to develop enhanced methodological approaches for further improving these cognitive effects prior to clinical translation. In terms of future research directions, the investigation of alternative individualised targeting methods (e.g., with neuroimaging) and comparing the relative cognitive effects with standardised targeting approaches (e.g., International 10–20 EEG system) will be important to determine the optimal targeting methodology. Stimulating alternative target sites other than the DLPFC (e.g., the posterior parietal cortex [44]) may produce enhanced cognitive effects. The investigation of the combination of rTMS with other interventions (e.g., cognitive training [45]) or adjunctive pharmacological agents (e.g., D-Cycloserine [46]) may additionally further enhance rTMS’s cognitive effects. Alternatively, research on other less focal brain stimulation methods currently in development, for example, gamma frequency sensory stimulation [47], or subcutaneous electrical stimulation [48], may have potential for producing superior cognitive effects relative to rTMS and other current psychological (e.g., cognitive training) and pharmacological approaches.

## 5. Conclusions

In conclusion, the current study showed no significant cognitive enhancing effects for cognitive flexibility following a single ‘offline’ iTBS session in healthy participants. This finding was despite the use of a novel method for individualising the stimulation site at the L-DLPFC that was previously demonstrated to be associated with the greatest modulating effects on cognitive flexibility when ‘online’ rTMS was applied. Future research is required to investigate alternative novel methodological approaches for administering rTMS for enhancing cognitive flexibility and other cognitive functions.

## Data Availability

The data presented in this study are available on request from the corresponding author. The data are not publicly available due to ethical restrictions.
